# Surgery

**Published:** 1860-05

**Authors:** S. W. Gross

**Affiliations:** Lecturer on Surgical Anatomy and Operative Surgery, Philadelphia


OR, BI-MONTHLY NOVELTIES IN MEDICAL, PATHOLOGICAL, SURGICAL, OBSTETRICAL, PHYSIOLOGICAL, AND CHEMICAL SCIENCE.

### SURGERY.

BY S. W. GROSS, M.D.,

LECTURER ON SURGICAL ANATOMY AND OPERATIVE SURGERY, PHILADELPHIA.

I.—*On Scalds of the Larynx.* By PHILIP BEVAN, M.D., Fellow and Professor of the Royal College of Surgeons, Ireland, etc. (Dublin Quarterly Journal of Medical Science, February, 1860.)

DR. BEVAN, in this valuable paper, reports four cases of scalds of the larynx, produced in young children by drinking boiling fluids from the spout of a kettle, and his object in doing so is to recommend for further trial a new plan of treatment in preference to the very unsatisfactory operation of tracheotomy. In regard to this procedure he shows that its fatality does not arise from delay, as in the majority of reported cases it was performed in less than seven hours after the occurrence of the accident, and most of the cases died, although they seemed to be improved by the operation for a short time. The author divides the *symptoms* produced by scalds of the larynx into three stages; and without entering into these in full, we will merely state that in the first stage the mouth and fauces are alone affected, and the respiration unembarrassed. These symptoms may be so mild as to mislead the practitioner, and from want of attention the patient may be lost. The second stage is characterized by laryngitis, giving rise to œdema of the glottis and incipient congestion of the lungs. In the third stage congestion of the brain and engorgement of the lungs are added to the previous symptoms.

The *treatment*, which is antiphlogistic, is commenced with an emetic, followed by a cathartic enema, and the application of a few leeches to the upper portion of the sternum, the number being regulated by the strength of the child. Should signs of the second stage appear, the system is brought as rapidly as possible under the influence of mercury, by the internal administration of calomel and inunctions with the blue ointment; the severity of the symptoms, the age and strength of the patient influencing the amount of the mineral employed. Leeches, which act by diminishing congestion of the brain, are to be repeated every three or four hours, care being taken to watch their effects. In these four cases, as soon as the mercury had made an impression on the system, and the green stools were produced, the symptoms abated and the child recovered; the lungs being first relieved, next the brain, and lastly the larynx. Although this treatment has been instituted in only four cases, yet as its results were uniformly successful, this line of practice should awaken the attention and consideration which the disease calling for it most unquestionably demands. All of these patients were affected with œdema of the glottis, erect and hard epiglottis, stridulous respiration, fixed pupils, pale, bloated features, cold surface, congested lungs, and incipient coma, symptoms which are considered sufficiently bad to justify a resort to tracheotomy. The operation may be tried in extreme cases, when dissolution seems to be immediate; but Dr. Bevan believes if the antiphlogistic treatment be conducted sufficiently rapidly so as to stop or retard the progress of the second stage, and ward off injury to the brain and lungs, it will be far more successful than tracheotomy.

[As bearing upon this subject we find, in the *British Medical Journal* for January 14, 1860, a report of Dr. Sloane, of Leicester, England, upon six cases of scalds of the glottis, five of which recovered under the use of antimony and calomel, which were given as soon as the accident happened, irrespective of the presence or absence of symptoms of implication of the lungs. In the fatal case, tracheotomy was performed, and in this patient the difficulty of breathing was not so great as in one who was cured. Mr. Wright, an experienced surgeon of Nottingham, states that tracheotomy for scalds of the larynx almost uniformly proves fatal, and he is in favor of the treatment by leeches, calomel, and antimony. In the *Medical Times and Gazette* for October, 1859, will be found a statistical report of fourteen cases of the same affection, occurring in England, in which tracheotomy was performed, the result being eleven deaths, notwithstanding the operation was resorted to in from four to seven hours after the accident. We, therefore, find that of ten cases of scalds of the larynx, treated by the antiphlogistic plan, nine recovered, the fatal one having been tracheotomized. Add to the English cases of tracheotomy in the same disease, the one of Dr. Sloane, and another one which came to his knowledge, but which he did not treat, and we have sixteen cases, thirteen proving fatal and three successful. In one of the cases of recovery, it would seem doubtful, from the peculiarity of its history, whether the glottis was scalded at all, thus making the proportion of recoveries only one in seven and a half.]

II.—*Selections from a Report on Ovariotomy.* Read before the Kentucky State Medical Society, at its Annual Meeting, at Louisville, April, 1857. By J. TAYLOR BRADFORD, M.D., of Augusta, Kentucky. (Reprint from the *Louisville Medical News*, 1859; pp. 57.)

The author of this report first discusses the operation of ovariotomy and its principal performers, and then enters upon the diagnosis of ovarian tumors and other diseases with which they are liable to be confounded. Letters are produced from various surgeons, the opinion of all of whom is entitled to respect, who coincide with Dr. Bradford as to the propriety of operative measures in suitable cases of this class of affections.

One of the greatest dangers in ovariotomy is the occurrence of hemorrhage, and, as is shown by the statistical tables of Dr. Lyman and Dr. Atlee, twenty-five per cent. of the cases die from this cause. The author refers this accident to the fact of the pedicle of the tumor being on the stretch when the ligature is applied, and in one case, when turning the stump of the pedicle to see if it were bleeding, he observed a part of it contracting within the ligature. "The pedicle, but more particularly the ligament of the ovary, is very extensible and elastic. If the tumor be lifted out with much force, or by any movement which places the pedicle on the stretch, so much so that it does not contract before the ligature is applied, that part of it which is most extensible when it does contract is apt to slip through the ligature, and still, without close examination, look as though all was right."

In advocating the operation in suitable cases, Dr. Bradford remarks, that it should be avoided when the tumor is small and not affecting the patient's constitution, as well as in those cases in which the tumor is so large, and the general health so much involved that it would be hazardous to operate.

Attached to this paper is a collection of all the cases, thirty in number, that have been operated upon in Kentucky; but in many the details are meagre, and render them incomplete in point of statistical interest. Of the thirty cases, nineteen recovered and eleven died, and in twenty-five of these was the operation completed. Of the remaining five, in which it was abandoned, two recovered and three proved fatal, the cause of failure in two cases, in which it is mentioned, being in one peritoneal adhesions, and in the other adhesions to the bladder and uterus. In four cases only is the cause of death stated, three being from peritonitis and one from hemorrhage. In twelve patients adhesions existed, and in but two was the short incision practiced. The disease began in one subject at the age of nine years, or three years before the establishment of the menstrual function; in one case the patient had ascites, for which she had tapped herself nineteen times; and being operated upon, no tumor was found, but a mass of intestines glued together by old adhesions. Finally, in one case the ligature did not come away until the eleventh month.

In a note appended to his report, Dr. Bradford states that up to November, 1859, he had operated upon or been jointly connected with eleven cases of ovarian tumor. All of these proved successful, with one exception, and that was a case of fibrous tumor of the uterus. Ten of these patients are still alive, and two have each had two children. The smallest tumor weighed twenty-four pounds, the largest sixty pounds; the youngest patient being single, and nineteen years of age, the oldest married, and sixty years old. Two of these cases, moreover, seem to have been unsuited for the operation.

Dr. Bradford has, therefore, attained a remarkable and unparalleled success, and we are certainly not astonished to find him so warm an advocate of a procedure which has been abused more than any other capital operation of surgery.

III.—*On Polyps of the Œsophagus.* By PROF. MIDDELDORPFF. (Vierteljahrschrift für die Praktische Heilkunde, vol. lxii. part ii. p. 50, 1859.)

Polyps of the œsophagus, strictly so called, in which the pedicle is attached to some portion of this canal, are of very rare occurrence, and the cause of their formation is very obscure. Frerichs is of the opinion that they are produced by obstruction and enlargement of the mucous glands; whereas other pathologists imagine that they are due to relaxation and swelling of the mucous membrane, a view which is favored by the fact that these tumors sometimes form in the narrow space near the larynx, on a level with the fourth, fifth or sixth cervical vertebra, where the food must pass to reach the œsophagus, and where the lax mucous membrane is thrown into folds by the contraction of the circular muscular fibres. The former theory is, however, the most plausible. The polyp rapidly grows downward, being elongated and drawn in this direction by the movements of deglutition, which it constantly excites, and extends the structure from which it arises in such a manner as to form a fold constituting its stalk or pedicle. The parts around the tumor are in an inflamed condition and covered with a mucous or purulent discharge, and the polyp is liable to undergo various changes, as exudation, suppuration, and hemorrhage, resulting from great swelling, or contraction of the œsophagus, or destruction of its epithelium.

One of the most prominent *symptoms* of this affection is difficulty in deglutition, and as the tumor enlarges, it increases to a distressing extent, and is accompanied with pain, difficulty in breathing, and sensations of suffocation. Nutrition is interfered with; the patient has nausea, and in his efforts at vomiting the polyp may rise up in the throat or mouth, thus rendering the diagnosis easy. There is almost always a discharge of mucus, which may be mixed with blood or pus, or with both. In making an examination with a view to detect the presence of the tumor, a probang may prove useful, or, when it is seated far down, a wire noose may catch it. The diagnosis is, however, difficult when it can neither be felt nor seen, and if this be the case, and the tumor be not removed, death will ensue from starvation and impeded respiration.

In regard to the *treatment*, when the polyp is visible, it may be seized and twisted off, or it may be cut off with long, curved scissors; but these operations are liable to be followed by profuse hemorrhage, which it will be difficult to restrain. Professor Middeldorpff does not think that ligation alone is sufficient, but advises, after the ligature has been applied, direct removal by the galvanic cautery, in this way preventing suffocation, which might at any time take place. He cites a case of polyp of the œsophagus, removed by this procedure in January, 1853, which is the first instance where the galvanic cautery was employed. He considers œsophagotomy too dangerous to be advised. When the polyp is not visible, the treatment must be palliative, the object being to maintain respiration and nutrition. Should the breathing become materially interfered with, bronchotomy might be resorted to, and the stomach-pump may be used for the introduction of food, but gastrotomy or enterotomy are not justifiable.

IV.—*A Historical and Critical Examination of the Operation of Tracheotomy in Croup, with a Report of Fourteen Cases.* By A. L. Voss, M.D., Surgeon to the Canal Street Dispensary, New York. (New York Journal of Medicine, January, 1860, p. 30.)

Dr. Voss has contributed to surgical literature a very valuable and interesting paper on the operation of tracheotomy in membranous croup, and we shall refer to it here in connection with the reports on the same subject of Mr. Conway Evans and Mr. James Spence, more in reference to statistical facts in regard to the propriety of the operation than to any other points contained in their contributions.

In the treatment of croup the knife is employed most generally as a dernier resort, when it is, with some exceptions, too late to be of much avail; and surgeons are now agreed that the operation should be performed when suffocation is imminent, to admit of the free access of air to the lungs. Dr. Voss does not wait until the patient is actually suffocating, but prefers operating when obstruction of the larynx is giving rise to commencing asphyxia, provided this be not relieved by an emetic. We cannot, however, follow the author in his interesting details, but will consider the results of the operation of tracheotomy in croup, as published in a few papers of the past year and early portion of the present year.

Dr. Voss has operated on fourteen patients, their ages varying from twenty months to five years, with five recoveries and nine deaths.

In the *Edinburgh Medical Journal* for February, 1860, Mr. Spence reports thirteen cases of croup in which he has operated in the last suffocative stage, the result being six cures to seven deaths. Mr. Evans read a paper before the Medical and Chirurgical Society of London, and which may be found in the *Medical Times and Gazette*, August 27, 1859, in which he reports four cases of croup and two of diphtheria (?) occurring in patients from two to ten years of age, the operation being followed by only one recovery. The same journal for October 15, 1859, contains the results of tracheotomy in croup, occurring in the different hospitals of London. The operation was performed as a dernier resort on fifteen patients, whose ages varied from thirteen months to six years, and only four recovered.

In the Transactions of the New York Academy of Medicine, as reported in the *New York Journal of Medicine*, March, 1860, will be found the statement of Dr. James Minor, in which he says that he has operated for croup six times, and has had two successful cases.

We thus gather from recent papers reports of fifty-four cases of membranous croup in which tracheotomy was performed, with the result of eighteen recoveries and thirty-six deaths, the proportion being two deaths to one cure. Dr. Voss's article contains interesting statistics, extending over several years, of a large number of cases, and when added to the cases above reported, we find we have 1249 operations with 294 recoveries and 955 deaths, or $3\frac{1}{4}$ deaths to 1 cure.

V.—*On Tibio-Tarsal Amputation.* By M. MICHAUX, Professor of Clinical Surgery at the University of Louvain. (Presse Médicale Belge, No. 33, 1859; and British and Foreign Review, January, 1860.)

This paper was presented to the Brussels Academy of Medicine by M. Michaux, with the object of comparing tibio-tarsal amputation with supra-malleolar amputation and amputation at the place of election. The conclusions of the author are contained in the following summary:—

1. Tibio-tarsal amputation is an operation properly admitted into surgical practice. 2. It should replace supra-malleolar amputation whenever the lesions which render it necessary are limited to the constituent parts of the foot, to the upper part of the malleoli, or even to the articular surface of the tibia. 3. It should only be performed when a flap can be formed of the soft parts of the heel, so that these may constitute a mattress for the bones. 4. If the soft parts of the heel are destroyed, or have undergone such alteration as to be unfitted for the purposes of a flap, the tibio-tarsal amputation should be renounced, the supra-malleolar operation being, as a general rule, substituted for it. 5. The best mode of performing tibio-tarsal amputation is by Syme's procedure, as modified by Jules Roux, of Toulon, taking, however, the precaution of preserving less integuments at the external side of the articulation (a modification of the author's) than is done by the latter surgeon, cutting the tendo-achillis sufficiently high, and excising the posterior tibial nerve, as recommended by M. Verneuil. 6. Pirogoff's operation may be had recourse to when the calcaneum is entirely healthy, or has undergone but little alteration at its anterior part. 7. Tibio-tarsal amputation is more difficult, more dangerous, and more slow in its after-treatment, than supra-malleolar amputation; but standing and walking being performed directly on the stump, are better executed after the former than after the latter. 8. The prothesis after tibio-tarsal amputation is far more simple and less expensive than that employed after supra-malleolar amputation. Syme's boot constitutes an excellent apparatus. 9. The best procedure for supra-malleolar amputation consists in forming an anterior flap, which, falling by its own weight, covers the bones. This is the elliptical operation, with the anterior flap of M. Poupart. 10. A procedure similar to that described by Baudens for disarticulation of the knee, should be adopted in amputation at the place of election, whenever the lesions admit of the preparatory mode being chosen. 11. Amputation at the place of election is more difficult and far more dangerous than supra-malleolar amputation, but walking is more easily performed after the former than after the latter.

12. To sum up. (*a*) Whenever the pathological condition allows of the surgeon choosing the place of amputation, he should prefer the tibio-tarsal in young, strong subjects endowed with a tolerably good constitution, whatever may be their position, sex, or profession. (*b*) Supra-malleolar amputation is suitable for aged and enfeebled subjects, who are called upon by their position to walk but little, and can obtain a good artificial limb which assumes nearly the normal form of the extremity. (*c*) Amputation at the place of election should be preferred to the supra-malleolar for young subjects, and especially children, who almost always recover after amputation. Also in strong subjects of a good constitution, to whom it is of greater importance to walk well than to disguise their mutilation, and whose position in life obliges them to undertake laborious occupations.

VI.—*On Tic Douloureux, the Painful Affection of the Face, with a New Operation for its Cure.* By J. M. CARNOCHAN, M.D., Professor of Surgery in the New York Medical College. (American Medical Monthly, Feb. 1860; p. 142.)

In this paper, presented to the Medico-Chirurgical College of New York, Professor Carnochan gives his views of the seat, pathology, and treatment of neuralgia of the face, and describes his new operation for exsecting the superior maxillary division of the fifth pair of nerves as far as the foramen rotundum of the sphenoid bone; his previous operation consisting in the removal of Meckel's ganglion and the trunk of the nerve beyond it.

Considering the true seat of the disease to be in the whole or some part of the trunk of the nerve in front of the ganglion of Gasser, the author proposes to relieve it by exsecting the nerve on the cerebral side of Meckel's ganglion. For this purpose an incision is commenced upon the middle of the zygomatic arch, opposite the spheno-maxillary fossa, and carried forward and slightly downward to a point a little below the infra-orbital foramen, and it is further extended so as to divide entirely the tissues of the cheek and lip midway between the median line and the commissure of the mouth. The flap is now dissected from the malar and upper jaw bones, and the nerve is to be sought for as it emerges from below the orbit; and being found and isolated, the foramen and lower border of the orbit are completely exposed. A small trephine is then applied just below the foramen, and a portion of the anterior wall of the antrum is removed with the lower part of the malar and the outer portion of the superior maxilla connected with it. The cavity of the antrum being now fully exposed by removing its outer wall, the nerve is detached from before backward by breaking down the wall of the infra-orbital canal, care being taken to avoid injury to the soft parts of the orbit. The next step consists in removing the portion of the nerve running through the spheno-maxillary fossa and with it the spheno-palatine ganglion. The lower jaw must be held firmly and depressed, and the tissues upon the posterior wall of the antrum being pushed backward, the fossa containing the nerve and internal maxillary artery is brought into view. Avoiding the latter, the nerve is pulled downward and isolated from the other tissues, and the foramen rotundum is to be ascertained by tracing with the finger the anterior border of the external pterygoid plate upward to its junction with the angle formed by the body and great wing of the sphenoid bone, and passing inward from the upper part of this angle for about two lines. With a blunt hook the nerve is still further detached where it emerges from the foramen, and gentle traction being made on it, it is severed at the base of the skull. The ganglion of Meckel can now be removed.

This paper is illustrated by the case of the man Forbes, who in the course of seven years and a half was the subject of thirteen operations for a very aggravated form of neuralgia. In October, 1857, Dr. Carnochan removed a part of the second branch of the fifth pair, and with it the spheno-palatine ganglion, an operation which relieved the patient for twelve months. In June, 1859, the patient again requested an operation, when the same external incisions were made as above described, the remaining portion of the nerve was found and divided at its point of emergence from the foramen rotundum. The result has been satisfactory up to the time of the report, about seven months after the operation.

The views of the author in relation to severe facial neuralgia are contained in the following propositions:—

1. That the second branch of the fifth pair, extending from the ganglion of Gasser to the infra-orbital foramen, has two peripheries: one, formed by the terminal branches of the trunk, given off along its course, to the superficial parts of the face; the other, by the terminal branches emanating from the ganglion of Meckel.

2. That in cases of severe *tic douloureux* (the *dolor crucians facei* of Fothergill) the seat of the disease is in a portion of the trunk of the nerve, or in the entire trunk, between the ganglion of Gasser and the foramen infra-orbitale, including that part embraced by the foramen.

3. That the trunk of the nerve being injured or diseased, pain is felt at its periphery, as well as in the part morbidly affected.

4. That impressions, acting upon the periphery of the nervous trunk, will be reflected upon the trunk, and give rise to paroxysms of neuralgic pain.

5. That the ganglion of Gasser, or the *common trunk* of the fifth pair, cannot be the seat of the disease, because experiments upon living animals, and pathological facts derived from post-mortem examination, demonstrate that when this ganglion and the trunk of the fifth pair are destroyed or injured, the eye of the corresponding side becomes destroyed from defective nutrition, and also that the other organs of special sense manifest symptoms of functional disorder.

6. That the encephalic strands of the fifth pair, on the cerebral side of the *common trunk*, cannot be the seat of the disease; as in such condition of the brain there would be symptoms denoting cerebral disturbance or disease, which never exist in tic douloureux.

7. That division of the nerve externally to the infra-orbital foramen, or anterior to the diseased portion of the trunk, will not effect a cure; because the point of disease being still left, the morbid sensibility is referred to the locality of the periphery, although that has been removed or insulated.

8. That when only a portion of the trunk of the nerve is removed, anterior to the ganglion of Meckel, the remaining portion may become affected with the disease, and the symptoms be renewed with the same severity as before the operation.

9. That the only operation which will cure the disease is the exsection of the nerve on the cerebral side of the ganglion of Meckel; because, first, the diseased part will thus be removed; second, because the two peripheries of the nerve must thus be insulated from the encephalon; third, because the influence of the ganglion of Meckel, in supplying morbid nervous sensibitity, is destroyed; fourth, because the sensibility of the two peripheries of the nerve is obliterated, and consequently external impressions cannot be reflected or transmitted.

10. That there is a possibility of the neuralgia returning for a time, even after the exsection of the trunk beyond the ganglion of Meckel, from disease attacking the small portion of the nerve still remaining in front of the ganglion of Gasser, or from pressure upon it, resulting from osteitis and contraction of the foramen rotundum; the pain being referred, as already explained, to the original seat of the periphery.

11. That in such a case, however, the stump of the nerve, whether diseased or compressed by the circumference of the foramen rotundum, would be placed under circumstances leading to atrophy or resolution; and that the disease, existing for a short time from such causes, would eventually subside.

12. That the three trunks of the fifth or trifacial nerve, emanating from the ganglion of Gasser, and supplying in their aggregate the general sensibility to the face, when affected by neuralgia, are to be subjected alike to the same rules in regard to the etiology, pathology, and treatment.

VII.—*Intra-Uterine Fracture of the Clavicle.* By WILLIAM B. ATKINSON, M.D. (Medical and Surgical Reporter, March 17, 1860.)

The patient was delivered naturally, after an easy labor, of a good-sized male child, without the attendance of a physician. A few days after, having taken upon herself the task of washing the infant, she detected a projection on the left side, between the shoulder and sternum. Upon an examination, the presence of a perfectly consolidated fracture of the clavicle was ascertained, the apex of the angle of junction pointing upward. From the fact of so short a time having elapsed from the birth of the child, and the complete union at the point of fracture, it was evident that the solution of continuity must have taken place some weeks prior to the completion of pregnancy. The mother had, some three or four weeks before her confinement, received a violent blow in her left side from the edge of a door.

